# Risk factors for ectopic pregnancy: a multi-center case-control study

**DOI:** 10.1186/s12884-015-0613-1

**Published:** 2015-08-22

**Authors:** Cheng Li, Wei-Hong Zhao, Qian Zhu, Shu-Jun Cao, Hua Ping, Xiaowei Xi, Guo-Juan Qin, Ming-Xing Yan, Duo Zhang, Jun Qiu, Jian Zhang

**Affiliations:** Department of Obstetrics and Gynecology, International Peace Maternity and Child Health Hospital, School of Medicine, Shanghai Jiao Tong University, Shanghai, 200030 China; Institute of Embryo-Fetal Original Adult Disease Affiliated to Shanghai Jiao Tong University School of Medicine, Shanghai, 200030 China; Department of Obstetrics and Gynecology, Songjiang Central Hospital, Shanghai, 201600 China; Department of Obstetrics and Gynecology, Songjiang Maternity and Child Health Hospital, Shanghai, 201620 China; Department of Obstetrics and Gynecology, Shanghai First People’s Hospital, Shanghai Jiao Tong University, Shanghai, 200080 China; Department of Obstetrics and Gynecology, Minhang Central Hospital, Shanghai, 201100 China

## Abstract

**Background:**

Ectopic pregnancy (EP) is the leading cause of maternal death during the first trimester of pregnancy. A better understanding of EP risk can help prevent its occurrence. We carried out a multi-center, large-sample, case-control study to evaluate the risk factors for EP in Shanghai, China.

**Methods:**

Women who were diagnosed with EP (*n* = 2411) and women with intrauterine pregnancies (*n* = 2416) were recruited from five hospitals in Shanghai, China. Information regarding the sociodemographic characteristics; reproductive, gynecological and surgical history; and previous and current use of contraceptives was collected from all participants. Odds ratios (ORs) and 95 % confidence intervals (CIs) were calculated and adjusted for potential confounding factors via multivariate logistic regression analysis.

**Results:**

The study revealed that the risk of EP was associated with the traditional risk factors including previous EP (Adjusted odds ratio [AOR] = 2.72, 95 % CI: 1.83–4.05), previous *Chlamydia trachomatis* infection (Adjusted OR = 3.18, 95 % CI: 2.64, 3.84), previous infertility (AOR = 2.18, 95 % CI: 1.66–2.88), previous adnexal surgery (AOR = 2.09, 95 % CI: 1.49–2.93), previous appendectomy (AOR = 1.64, 95 % CI: 1.13–2.37), and previous use of intrauterine devices (IUDs) (AOR = 1.72, 95 % CI: 1.39–2.13). Additionally, EP risk was increased following the failure of most contraceptives used in the current cycle including IUDs (AOR = 16.43, 95 % CI: 10.42–25.89), oral contraceptive pills (AOR = 3.02, 95 % CI: 1.16–7.86), levonorgestrel emergency contraception (AOR = 4.75, 95 % CI: 3.79–5.96), and female sterilization (AOR = 4 .73, 95 % CI: 1.04–21.52). Stratified analysis showed that *in vitro* fertilization and embryo transfer (IVF-ET) was the main risk factor for EP in women with tubal infertility (AOR = 8.99, 95 % CI: 1.98–40.84), although IVF-ET showed no association with EP in women with non-tubal infertility (AOR = 2.52, 95 % CI: 0.14–44.67).

**Conclusion:**

In addition to the traditional risk factors, IVF-ET and current IUD use play dominant roles in the occurrence of EP. Attention should be given to women with tubal infertility who have undergone IVE-ET treatment.

## Background

Ectopic pregnancy (EP) is the leading cause of maternal death during the first trimester of pregnancy, accounting for approximately 10 % of all pregnancy-related deaths [1]. It remains to be a condition presenting as a serious health problem for women of childbearing age [2]. It has been shown to reduce subsequent fertility and increase the chances of subsequent EP [3]. Over recent decades, there has been a rise in the incidence of EP [4].

There is extensive literature regarding the potential risk factors for EP. The identified risk factors for EP include age, previous EP, previous pelvic surgery, use of intrauterine devices (IUDs), female sterilization, history of pelvic inflammatory disease, history of infertility and smoking at the time of conception [5–12]. The increased awareness and knowledge on the risk factors for EP could enable an early and accurate diagnosis of the disease, resulting in a reduced need for surgery and fewer complications.

However, the study designs of previous researches focused on women not using contraception at the time of conception to explore the risk factors for EP comprehensively, which failed to evaluate the association between EP and contraceptive using in the current cycle of conception and might make the results in an overall ambiguity [7], because fertility intention might have a great impact on pregnancy outcome when study the risk factors for EP [13]. Furthermore, with the increased incidence of EP, and variance in population structure and regional differences, the risk factors of EP may have changed. Additionally, due to the increased incidence in infertility, currently approaches such as assisted reproduction technology (ART) are more widely used; therefore, their role and strength in the incidence of EP should be re-evaluated [14, 15]. Different from the previous study which investigated the risk factors for EP in women with planned pregnancy only in one hospital in Shanghai [16], the present was designed to conduct in five hospitals covering the population across urban and rural areas of Shanghai with a relatively good representation of the population, in order to comprehensively evaluate all the risk factors for EP among the general population, rather than those with planned pregnancy.

## Methods

### Study design and participants

This case–control study was conducted at five medical hospitals in Shanghai (two general hospitals and three maternity hospitals). The study protocol was approved by the Institutional Review Board of all the hospitals (International Peace Maternity and Child Health Hospital, Shanghai First People’s Hospital, Songjiang Central Hospital, Songjiang Maternity and Child Health Hospital, and Minhang Central Hospital). Written informed consents were obtained from all the study participants before they were interviewed.

From March 2011 and April 2013, women who had been diagnosed with EPs in the inpatient gynecology department of each hospital were interviewed as potential candidates for the case group (EP group). Women with intrauterine pregnancy (IUP) at the prenatal and family planning clinics of the same hospitals matched for age (±5 years), marital status and gestational age (±7 days) at a ratio of 1:1 were included in the study as controls. The inclusion criteria of the study subjects were as described in our previous study [17].

### Data collection and variable specification

The definitions of previous and current use of contraceptive methods have been described in our previous study [17].

All participants were interviewed via a questionnaire according to a standard protocol to obtain information on sociodemographic characteristics (age, marital status, education, birthplace, personal annual income, smoking and institutions); reproductive, gynecological and surgical history (including number of previous abortions, parity, history of previous EP, previous infertility, categories of infertility, ARTs applied in the current cycle of conception, previous Cesarean section, previous adnexal surgery, specific adnexal surgery, previous appendectomy); previous use of contraceptives (including levonorgestrel emergency contraception [LNG-EC]; IUDs; oral contraceptive pills [OCPs]; and other methods such as condoms, withdrawal method and calendar rhythm method), and current use of contraceptives (IUDs; OCPs; LNG-EC; female sterilization; and other methods such as condoms, withdrawal method and calendar rhythm method).

Serum *Chlamydia trachomatis* (CT) IgG antibodies were detected using enzyme-linked immunosorbent assay (ELISA; Beijing Biosynthesis Biotechnology, China) after collecting 5-mL blood samples from each participant.

### Statistical analysis

We examined the frequency distribution of each variable according to the case and control groups. Univariate logistic regression analysis was used to estimate the crude odds ratios (ORs) of each variable and their 95 % confidence intervals (CIs). The variables associated with EP by univariate analysis were included as candidates in the multivariable logistic regression analysis by stepwise selection with a SLENTRY level of 0.1 and SLSTAY level of 0.1.

When we explored the association between the risk of EP and ART, history of infertility was a major confounding factor. Thus, the study participants with history of infertility were divided into two strata, women with tubal infertility and those with non-tubal infertility. The association between risk of EP and each ART were analyzed in both strata. ORs and their 95 % CIs were calculated and adjusted for other potential confounding factors, including age (less than 20, 20–24, 25–30, 30–34, 35–39, or greater than 40 years of age), medical hospitals (1, 2, 3, 4 or 5), educational level (primary school or less, middle school, high school or college or higher degree), occupation (employed, self-employed or unemployed), previous EP (no or yes), serum CT IgG test (negative or positive), previous adnexal surgery (no or yes), previous appendectomy (no or yes), previous use of IUDs (no or yes), previous use of other contraceptive methods (no or yes), and current use of contraceptive methods (not used, OCPs, LNG-EC, IUDs, female sterilization or other contraceptive methods).

All statistical analyses were performed using SAS software, version 8.2 (SAS Institute, Inc., Cary, NC). All *p* values were calculated using two-sided tests. Differences between values were considered statistically significant at a *p* value of less than 0.05.

## Results

**Fig. 1:**
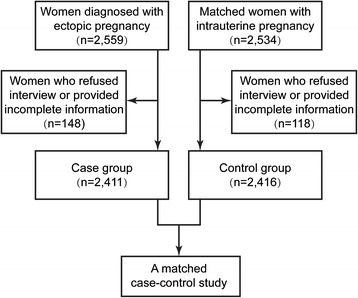
Recruitment profile of the case–control study

From the study subjects, 148 EP and 118 IUP patients either refused to participate in the interview or provided incomplete information in the questionnaire survey; these women were excluded from the study. Finally, 2411 EP patients and 2416 IUP controls were included in this study, and the response rate was 94.78 % (Fig. 1).

### Univariate analysis

Table 1 presents the distribution of sociodemographic characteristics between both the groups. EP patients were more likely to be born out of Shanghai (*p* < 10^−3^), have lower education attainment (*p* < 10^−3^), and be self-employed/unemployed (*p* < 10^−3^). However, smoking showed no relevance with EP (occasional smoker: OR = 1.10, 95 % CI: 0.77–1.59; regular smoker: OR = 1.47, 95 % CI: 0.95–2.28; *p* = 0.41). Due to the matching criteria of cases and controls in each hospital, there showed no significant difference in age (*p* = 0.16), marital status (*p* = 0.56) and institutions (*p* = 1.00) between the two groups.

**Table 1:** Socio-demographic characteristics of all enrolled participants

	EP		IUP		OR [95 % CI]	*p* value
	*n* ^a^	(%)	*n* ^a^	(%)		
Age (year)						
≤20	24	(1.00)	32	(1.33)	Reference	0.16
20–24	363	(15.06)	398	(16.47)	1.22 [0.70, 2.10]	
25–29	753	(31.23)	772	(31.95)	1.30 [0.76, 2.23]	
30–34	793	(32.89)	755	(31.25)	1.40 [0.82, 2.40]	
35–39	332	(13.77)	322	(13.33)	1.38 [0.79, 2.39]	
≥40	146	(6.06)	137	(5.67)	1.42 [0.80, 2.53]	
Marital status						
Married	2067	(85.80)	2088	(86.42)	Reference	0.56
Unmarried	342	(14.20)	328	(13.58)	0.92 [0.78, 1.08]	
Birth place						
Shanghai	698	(28.95)	775	(32.08)	Reference	<10^−3^
Outside of Shanghai	1713	(71.05)	1641	(67.92)	1.16 [1.03, 1.31]	
Education attainment						
Collage or above	1061	(44.01)	1378	(57.04)	Reference	<10^−3^
High school	314	(13.02)	280	(11.59)	1.46 [1.22, 1.74]	
Middle school	178	(7.38)	195	(8.07)	1.19 [0.95, 1.48]	
Primary school or lower	858	(35.59)	563	(23.30)	1.98 [1.73, 2.26]	
Occupation						
Employed	1682	(69.88)	1897	(78.58)	Reference	<10^−3^
Self-employed	262	(10.89)	184	(7.62)	1.61 [1.32, 1.96]	
Unemployed	463	(19.24)	333	(13.80)	1.57 [1.34, 1.83]	
Personal annual income (RMB)						
<50,000	1165	(48.32)	1093	(45.24)	Reference	0.13
50,000-100,000	777	(32.23)	841	(34.81)	0.87 [0.76, 0.99]	
> 100,000	469	(19.45)	482	(19.95)	0.91 [0.79, 1.06]	
Smoking^b^						
None smoker	2298	(95.31)	2294	(96.18)	Reference	0.41
Occasional smoker	63	(2.61)	57	(2.39)	1.10 [0.77, 1.59]	
Regular smoker	50	(2.07)	34	(1.43)	1.47 [0.95, 2.28]	
Institutions^c^						
1	1404	(58.23)	1409	(58.32)	Reference	1.00
2	276	(11.45)	274	(11.34)	1.01 [0.84, 1.21]	
3	291	(12.07)	293	(12.13)	1.00 [0.83, 1.19]	
4	272	(11.28)	272	(11.26)	1.00 [0.84, 1.21]	
5	168	(6.97)	168	(6.95)	1.00 [0.80, 1.26]	

*EP* ectopic pregnancy, *IUP* intrauterine pregnancy, *OR* odds ratio, *CI* confidence interval

^a^The sum does not necessarily equal the sample size for all variables because of missing data

^b^Occasional smoker: cigarette smoking more than 4 times a week, but a day on average less than 1 cigarette. Regular smoker: cigarette smoking more than 1 cigarettes per day, continuous or accumulated 6 months

^c^Center 1 = International Peace Maternity and Child Health Hospital; Center 2 = Shanghai First People's Hospital; Center 3 = Songjiang Central Hospital; Center 4 = Songjiang Maternity and Child Health Hospital; Center 5 = Minhang Central Hospital

Table 2 revealed the results of the analyses of crude association between the risk of EP and history of reproduction, gynecology and surgery. The occurrence of EP were showed to have a crude association with some factors including parity (once: OR = 1.14, 95 % CI: 1.02–1.30; more than twice: OR = 1.58, 95 % CI: 1.27–1.96), previous EP (OR = 6.67, 95 % CI: 5.04–9.11), previous CT infection (OR = 3.83, 95 % CI: 3.27–4.48), history of infertility (OR = 4.42, 95 % CI: 3.53–5.53), *in vitro* fertilization and embryo transfer (IVF-ET; OR = 5.01, 95 % CI: 1.53–16.44), previous adnexal surgeries (OR = 5.42, 95 % CI: 4.29–6.84), and previous appendectomy (OR = 1.67, 95 % CI: 1.21–2.31).

**Table 2:** History of reproduction, gynecology, and surgery of all enrolled participates

	EP		IUP		OR [95 % CI]	*p* value
	*n* ^a^	(%)	*n* ^a^	(%)		
Reproductive history						
Number of previous abortions						
0	873	(36.88)	930	(38.49)	Reference	0.71
1	763	(32.23)	756	(31.29)	1.08 [0.94, 1.23]	
2	485	(20.49)	497	(20.57)	1.04 [0.89, 1.21]	
≥ 3	246	(10.39)	233	(9.64)	1.13 [0.92, 1.38]	
Parity						
0	1143	(48.29)	1280	(52.98)	Reference	<10^−3^
1	994	(41.99)	973	(40.27)	1.14 [1.02, 1.30]	
≥ 2	230	(9.72)	163	(6.75)	1.58 [1.27, 1.96]	
Gynecologic history						
Previous EP						
No	2093	(86.81)	2363	(97.81)	Reference	<10^−3^
Yes	318	(13.19)	53	(2.19)	6.77 [5.04, 9.11]	
Serum Chlamydia trachomatis IgG test						
Negative	1648	(69.13)	2099	(89.55)	Reference	<10^−3^
Positive	736	(30.87)	245	(10.45)	3.83 [3.27, 4.48]	
Previous infertility						
No	2005	(83.26)	2286	(95.65)	Reference	<10^−3^
Yes	403	(16.74)	104	(4.35)	4.42 [3.53, 5.53]	
Categories of infertility						
No	2005	(83.26)	2286	(95.65)	Reference	<10^−3^
Tubal infertility	326	(13.54)	73	(3.05)	5.09 [3.92, 6.61]	
Non-tubal infertility	77	(3.20)	31	(1.30)	2.83 [1.86, 4.32]	
ARTs applied in the current cycle of conception^b^						
Spontaneous pregnancy	278	(68.98)	72	(69.23)	Reference	<10^−3^
IVF-ET	58	(14.39)	3	(2.88)	5.01 [1.53, 16.44]	
Other ARTs^c^	46	(11.41)	18	(17.31)	0.66 [0.36, 1.21]	
Chinese herb	21	(5.21)	11	(10.58)	0.49 [0.23, 1.07]	
Surgical history						
Previous cesarean section^d^						
No	691	(56.09)	624	(54.74)	Reference	0.79
Yes	541	(43.91)	516	(45.26)	0.95 [0.81, 1.11]	
Previous adnexal surgery						
No	1985	(82.33)	2322	(96.19)	Reference	<10^−3^
Yes	426	(17.67)	92	(3.81)	5.42 [4.29, 6.84]	
Specific adnexal surgery^e^						
Ovarian surgery	64	(15.02)	36	(39.13)	2.08 [1.38, 3.14]	<10^−3^
Surgery for EP	237	(55.62)	38	(41.30)	7.30 [5.15, 10.33]	
Tubal reconstructive surgery	90	(21.13)	14	(15.22)	7.52 [4.27, 13.25]	
Female sterilization	21	(4.93)	2	(2.17)	12.28 [2.88, 52.45]	
Reversal of tubal sterilization	14	(3.29)	2	(2.17)	8.19 [1.86, 36.07]	
Previous appendectomy						
No	2303	(95.84)	2346	(97.47)	Reference	0.01
Yes	100	(4.16)	61	(2.53)	1.67 [1.21, 2.31]	

*EP* ectopic pregnancy, *IUP* intrauterine pregnancy, *OR* odds ratio, *CI* confidence interval, *ART* assisted reproduction technology, *IVF-ET in vitro* fertilization and embryo transfer

^a^The sum does not necessarily equal the sample size for all variables because of missing data

^b^The number of women with history of infertile was used as the denomintor to calculate the percentage

^c^Other ARTs includes ovarian stimulation, intrauterine insemination, luteal phase support and combination of ovarian stimulation and luteal phase support

^d^The number of women having delivered a child was used as the denomintor to calculate the percentage

^e^The number of women experienced adnexal surgeries was used as the denomintor to calculate the percentage

In terms of the contraceptive experience (Table 3), previous IUD use was also associated with a higher risk of EP with an OR of 1.48 (95 % CI: 1.25–1.74), whereas previous use of other methods including condom, rhythm method and withdrawal method was associated with a lower risk (OR = 0.39, 95 % CI: 0.34–0.45). Furthermore, a crude association was found between current use of most contraceptives and risk of EP (OCPs: OR = 2.71, 95 % CI: 1.11–6.61; LNG-EC: OR = 2.79, 95 % CI: 2.27–3.43; IUDs: OR = 11.41, 95 % CI: 7.45–17.48; female sterilization: OR = 12.45, 95 % CI: 2.91–53.18).

**Table 3:** Previous and current use of contraception

	EP		IUP		OR [95 % CI]	*p* value
	*n* ^a^	(%)	*n* ^a^	(%)		
Previous use of contraceptives						
LNG-EC						
No	1283	(53.57)	1311	(54.74)	Reference	0.53
Yes	1112	(46.43)	1084	(45.26)	1.05 [0.94, 1.17]	
IUDs						
No	1993	(83.74)	2106	(88.27)	Reference	<10^−3^
Yes	387	(16.26)	280	(11.74)	1.48 [1.25, 1.74]	
OCPs						
No	2254	(94.47)	2295	(95.55)	Reference	0.06
Yes	132	(5.53)	107	(4.45)	1.25 [0.97, 1.63]	
Other contraceptive methods^b^						
No	829	(35.32)	421	(17.50)	Reference	<10^−3^
Yes	1518	(64.68)	1985	(82.50)	0.39 [0.34, 0.45]	
Current contraceptive methods						
No	1337	(55.87)	1585	(65.60)	Reference	<10^−3^
Other contraceptive methods^b^	447	(18.68)	653	(27.03)	0.81 [0.71, 0.93]	
OCPs	16	(0.67)	7	(0.29)	2.71 [1.11, 6.61]	
LNG-EC	341	(14.25)	145	(6.00)	2.79 [2.27, 3.43]	
IUDs	231	(9.65)	24	(0.99)	11.41 [7.45, 17.48]	
Female sterilization^c^	21	(0.88)	2	(0.08)	12.45 [2.91, 53.18]	

*EP* ectopic pregnancy, *IUP* intrauterine pregnancy, *OR* odds ratio, *CI* confidence interval, *IUDs* intrauterine devices, *LNG-EC* levonorgestrel emergency contraception, *OCPs* oral contraceptive pills

^a^The sum does not necessarily equal the sample size for all variables because of missing data

^b^Other contraceptive methods includes condom, rhythm method, withdrawal

^c^Women who received reversal of tubal sterilization (*n* = 16) are not included here

### Multivariate analysis

Table 4 shows the results of the multivariate analysis between the risk of EP and candidate risk factors. Poor education and occupation were found to be independently associated with the risk of EP. The results revealed that women with previous EP (adjusted OR [AOR] = 2.72, 95 % CI: 1.83–4.05), previous CT infection (AOR = 3.18, 95 % CI: 2.64–3.84), a history of infertility (AOR = 2.18, 95 % CI: 1.66–2.88), previous adnexal surgery (AOR = 2.09, 95 % CI: 1.49–2.93), and previous appendectomy (AOR = 1.64, 95 % CI: 1.13–2.37) were at a greater risk of having an EP. With regards to contraception, previous IUD use was found to slightly increase the risk of EP (AOR = 1.72, 95 % CI: 1.39–2.13), while previous use of other contraceptive methods including condom, rhythm method and withdrawal method were shown to protect women from the incidence of EP (AOR = 0.56, 95 % CI: 0.47–0.66). In addition, current use of most contraceptives was significantly correlated with the incidence of EP following contraceptive failure, and the risk varied across the different contraceptive methods (OCPs: AOR = 3.02, 95 % CI: 1.16–7.86; LNG-EC: AOR = 4.75, 95 % CI: 3.79–5.96; IUDs: AOR = 16.43, 95 % CI: 10.42–25.89; female sterilization: AOR = 4.73, 95 % CI: 1.04–21.52). Notably, among women with a history of infertility, those who resorted to IVF-ET in the current cycle of conception showed a higher risk of EP (AOR = 9.28, 95 % CI: 2.14–40.38) than those who got spontaneously pregnant, while the use of Chinese herbal medicine and other ART approaches was not associated with the risk of EP (Chinese herbal medicine: OR = 0.80, 95 % CI: 0.41–1.56; other ARTs: OR = 0.8, 95 % CI: 0.34–1.88).

**Table 4:** Multivariable logistic regression analysis predicting risk factors for ectopic pregnancy

	AOR [95 % CI]	*p* value
Education attainment		
Collage or above	Reference	<10^−3^
High school	1.21 [0.97, 1.50]	
Middle school	0.96 [0.74, 1.24]	
Primary school or lower	1.47 [1.23, 1.75]	
Occupation		
Employed	Reference	<10^−2^
Self-employed	1.30 [1.02, 1.66]	
Unemployed	1.33 [1.10, 1.62]	
Previous ectopic pregnancy		
No	Reference	<10^−3^
Yes	2.72 [1.83, 4.05]	
Serum Chlamydia trachomatis IgG test		
Negative	Reference	<10^−3^
Positive	3.18 [2.64, 3.84]	
Previous infertility		
No	Reference	<10^−3^
Yes	2.18 [1.66, 2.88]	
ART applied in the current cycle of conception		
Spontaneous pregnancy	Reference	<10^−3^
IVF-ET	9.28 [2.14, 40.38]	
Other ARTs^a^	0.80 [0.34, 1.88]	
Chinese herb	0.80 [0.41, 1.56]	
Previous adnexal surgery		
No	Reference	<10^−3^
Yes	2.09 [1.49, 2.93]	
Previous appendectomy		
No	Reference	0.01
Yes	1.64 [1.13, 2.37]	
Previous use of IUDs		
No	Reference	<10^−3^
Yes	1.72 [1.39, 2.13]	
Previous use of other contraceptive methods^b^		
No	Reference	<10^−3^
Yes	0.56 [0.47, 0.66]	
Current contraceptive methods		
No	Reference	<10^−3^
Other contraceptive methods^b^	1.22 [0.99, 1.44]	
OCPs	3.02 [1.16, 7.86]	
LNG-EC	4.75 [3.79, 5.96]	
IUDs	16.43 [10.42, 25.89]	
Female sterilization	4.73 [1.04, 21.52]	

*AOR* adjusted odds ratio, *CI* confident interval, *ART* assisted reproduction technology, *IVF-ET in vitro* fertilization and embryo transfer, *IUDs* intrauterine devices, *LNG-EC* levonorgestrel emergency contraception, *OCPs* oral contraceptive pills

^a^Other ARTs includes ovarian stimulation, intrauterine insemination, luteal phase support and combination of ovarian stimulation and luteal phase support

^b^Other contraceptive methods includes condom, rhythm method, withdrawal

Table 5 presents a stratified analysis of the association between EP risk and the ARTs applied in the current conception cycle, according to the different categories of infertility. In women with tubal infertility, IVF-ET was shown to significantly increase the risk of EP (AOR = 8.99, 95 % CI: 1.98–40.84). However, there were no significant associations between the risk of EP and IVF-ET among women with non-tubal infertility (AOR = 2.52, 95 % CI: 0.14–44.67). The risk of EP among women using Chinese herbs and other ARTs remained the same as that before stratification.

**Table 5:** The association between EP and the mode of current pregnancy among women with tubal infertility or non-tubal infertility

	Tubal infertility^a^					Non-tubal infertility^b^				
	EP		IUP		AOR [95 % CI]^d^	EP		IUP		AOR [95 % CI]^d^
	*n* ^c^	(%)	*n* ^c^	(%)		*n* ^c^	(%)	*n* ^c^	(%)	
ARTs applied in the current cycle of conception										
Spontaneous pregnancy	223	(68.41)	50	(68.49)	Reference	55	(71.43)	22	(70.97)	Reference
IVF-ET	55	(16.87)	2	(2.74)	8.99 [1.98, 40.84]	3	(3.90)	1	(3.23)	2.52 [0.14, 44.67]
Other ARTs^e^	29	(8.9)	12	(16.44)	0.59 [0.25, 1.39]	17	(22.08)	6	(19.36)	0.94 [0.22, 3.98]
Chinese herb	19	(5.83)	9	(12.33)	0.78 [0.31, 2.01]	2	(2.60)	2	(6.45)	0.35 [0.03, 4.50]

*EP* ectopic pregnancy, *IUP* intrauterine pregnancy, *AOR* adjusted odds ratio, *CI* confident interval, *IVF-ET in vitro* fertilization and embryo transfer, *ART* assisted reproduction technology

^a^This analysis was restricted to 399 people with tubal infertility

^b^This analysis was restricted to 108 people with non-tubal infertility

^c^The sum does not necessarily equal the sample size for all variables because of missing data

^d^Odds ratios were adjusted for age, institutions, education attainment, occupation, previous EP, serum CT IgG test, previous adnexal surgery, previous appendectomy, previous use of IUDs, previous use of other contraceptive methods and current contraceptive methods

^e^Other ARTs includes Ovarian stimulation, intrauterine insemination, luteal phase support and combination of ovarian stimulation and luteal phase support

## Discussion

Here, IVF-ET and current IUD use were found to be high-risk factors associated with the incidence of EP in China. It has been suggested that tubal factor infertility rather than IVF-ET contributes to EP risk in women who undergo IVE-ET. Furthermore, other traditional risk factors were still found to be associated with the incidence of EP.

The traditional risk factors for EP such as history of previous EP, previous adnexal surgery, previous appendectomy and CT infection have been well described elsewhere [1, 3, 6, 10]. It is not surprising that the results of the current study are identical to previous reports of increased subsequent risk of EP by these traditional risk factors. This finding indicated that traditional risk factors still played a major role in the occurrence of EP.

Contraceptive failure is considered to be an important factor associated with the increased incidence of EP [18]. Due to the national family planning policy, there may be a difference in the contraception preferences in China [19]. All methods of contraception can effectively reduce the number of intrauterine and ectopic pregnancies. However, from our findings, when pregnancies occur as a result of contraceptive failure, the risk of ectopic pregnancy is significantly increased in women who become pregnant after tubal sterilization or after using IUDs, OCPs and LNG-EC. This finding from the present study was identical to the results from a meta-analysis [20]. Previous studies indicated that progesterone and its analogue, LNG, could effectively inhibit human tubal activities [21, 22], which has been considered as the main cause of impaired embryo-tubal retention and implantation [23]. Although we failed to obtain the information on the type of OCPs, women following OCPs failure still should be aware of the possibilities of EP.

The results of this study were consistent with those of previous studies, which showed that a history of infertility was a risk factor for EP [7, 9, 24]. It has been acknowledged that IVF-ET is a valuable treatment for infertility, especially for the treatment of tubal infertility. Ever since the first pregnancy conceived following IVF-ET treatment in 1976 was an ectopic one [25], the association between EP risk and ARTs has been debated [26]. The incidence of EP following ARTs, especially IVF-ET, has been reported to reduce since 2001 [27]. However, another study investigated the incidence of EP in 128,314 pregnancies following ARTs according to the presence or absence of tubal infertility, and concluded that the incidence of EP following ARTs was higher in women with tubal infertility than in women without tubal infertility [28]. In addition, Strandell et al. suggested that tubal infertility was the most prominent risk factor for EP following IVF-ET [29]. However, our previous study failed to explore whether ART have an influence on the risk of EP, due to the small number of women with non-tubal infertility becoming pregnant with ARTs [16]. Based on this large multi-center study, the stratification analysis indicated that women with tubal infertility and not those with non-tubal infertility were at a greater risk of EP when they underwent IVF-ET in their current conception cycle. Therefore, the present study indicated that it was IVF-ET that contributed to the risk of EP for women who underwent IVF-ET.

The present study has some limitations. Data collection in this study was based on patients’ self-evaluation; hence, we were unable to obtain additional information on the types of OCPs, sterilization methods and IUDs for further study. In addition, a limited number of women with non-tubal infertility underwent IVF-ET to become pregnant; thus, further prospective cohort studies are needed to verify our findings on the association between EP and IVF-ET among women with non-tubal infertility. Moreover, our data did not cover the technical and qualitative aspects of the IVF procedure (e.g., the stimulation protocols, endometrial and ovarian responses, embryo quality, transfer technique, number of embryos transferred and use of luteal support). Together, all these aspects may contribute to the risk of EP in women with IVF-ET [29]. As a hospital-based case-control study, recall and selection bias must be acknowledged. The multi-centered design carried out across five hospitals covering the urban and rural areas of Shanghai and the large sample size are the strengths of this study, and helped recruit a relatively good representation of the population thereby minimizing selection bias.

## Conclusion

In this large multi-center case-control study, the risk of EP still show correlation with some traditional risk factors including previous EP, previous CT infection, previous infertility, previous adnexal surgery, previous use of IUDs and current use of IUDs, OCPs, LNG-EC and female sterilization. Although IVF-ET has been associated with an increased risk of EP incidence among women with a history of infertility, this was only observed among women with tubal infertility and not in women with non-tubal infertility. In general, physicians should pay attention to suspected EP cases with exposure to some traditional risk factors. Furthermore, attention of EP should also be paid to pregnant women following IVF-ET, particularly among tubal infertility cases.

## Abbreviations

EP: Ectopic pregnancy
IUDs: Intrauterine devices
ART: Assisted reproduction technology
IUP: Intrauterine pregnancy
LNG-EC: Levonorgestrel emergency contraception
OCPs: Oral contraceptive pills
CT: *Chlamydia trachomatis*
ELISA: Enzyme-linked immunosorbent assay
OR: Odds ratio
CI: Confidence interval
IVF-ET: *In vitro* fertilization and embryo transfer
